# 超高效液相色谱-串联质谱法测定人血清中22种有机紫外吸收剂

**DOI:** 10.3724/SP.J.1123.2024.10031

**Published:** 2025-06-08

**Authors:** Dejun BAO, Zhuangzhuang FENG, Xu ZHANG, Qi SUN, Zhuona ZHANG, Xiaojian HU, Ying ZHU, Xiao LIN

**Affiliations:** 中国疾病预防控制中心环境与健康相关产品安全所，中国疾病预防控制中心环境与 人群健康重点实验室，北京 100021; China CDC Key Laboratory of Environment and Population Health，National Institute of Environmental Health，Chinese Center for Disease Control and Prevention，Beijing 100021，China

**Keywords:** 超高效液相色谱-串联质谱, 有机紫外吸收剂, 血清, 蛋白质沉淀技术, 高效脂质去除技术, 人体生物监测, ultra performance liquid chromatography-tandem mass spectrometry **（**UPLC-MS/MS**）**, organic ultraviolet absorbents （OUVs）, serum, protein precipitation technology （PPT）, efficiency lipid removal technology （ELR）, human biomonitoring

## Abstract

本研究将蛋白质沉淀技术、高效脂质去除技术与超高效液相色谱-串联质谱（UPLC-MS/MS）技术结合，建立了一种同时测定人血清中22种有机紫外吸收剂（OUVs）的方法。采用Acquity BEH C_18_色谱柱（100 mm×2.1 mm，1.7 μm）进行分离，分别以甲醇-水和甲醇-0.1%氨水作为流动相进行梯度洗脱。质谱分析采用正、负离子电喷雾电离（ESI^+^/ESI^‒^）源，在多反应监测（MRM）模式下扫描，稳定同位素内标法定量。实验结果表明，22种目标化合物在各自的线性范围内线性关系良好，相关系数（*r*）均≥0.999 3，方法检出限（MDL）为0.02~0.48 ng/mL，方法定量限（MQL）为0.02~1.60 ng/mL。在低、中、高3个加标水平下，22种目标分析物的加标回收率为79.9%~136.1%，日内精密度为1.5%~25.4%，日间精密度为0.6%~23.5%。采用稳定同位素内标法进行校正后，22种目标分析物在胎牛血清中的基质效应为83.0%~119.9%。应用该方法测定110份人血清样本，结果显示，除3-亚苄基樟脑（3-BC）、2-（2*H*-苯并三唑-2-基）-4-甲基-6-（2-丙烯基）苯酚（UV-9）、4-甲基苄亚基樟脑（4-MBC）、2，2′-羟基-4-甲氧基二苯甲酮（BP-8）和2，4-二羟二苯甲酮（BP-1）未检出外，其余17种目标分析物均有检出，检出率为0.9%~65.5%，检出水平为<MQL~11.7 ng/mL。该方法操作简单、便捷，灵敏度高且定量准确，适用于人血清中22种OUVs的测定。

## Abstract (en)

In this study， a method for the simultaneous determination of 22 organic ultraviolet absorbers （OUVs） in human serum was established by combining protein precipitation technology （PPT）， efficiency lipid removal technology （ELR） and ultra performance liquid chromatography-tandem mass spectrometry （UPLC-MS/MS）. The OUVs include five benzophenone compounds， five benzotriazole compounds， two cinnamate ester compounds and their three metabolites， two salicylate compounds， two camphor derivative compounds， one triazine compound， one dibenzoylmethane compound and one amino aminobenzoic acid derivative compound. Chromatography was performed using an Acquity BEH C_18_ column （100 mm×2.1 mm， 1.7 μm）， gradient elution was carried out using methanol-water and methanol-0.1% ammonia water as the mobile phases. Compounds were detected in both positive and negative electrospray ionization （ESI^+^/ESI^‒^） modes using multiple reaction monitoring （MRM）， and quantified using stable-isotope internal standards. The experimental results showed that the 22 target compounds exhibited good linear relationships within their respective linear ranges， with the correlation coefficients （*r*）≥0.999 3. The method detection limits （MDLs） ranged from 0.02 to 0.48 ng/mL， and the method quantification limits （MQLs） ranged from 0.02 to 1.60 ng/mL. At the three spiked levels of low， medium and high， the spiked recoveries of the 22 target analytes ranged from 79.9% to 136.1%， the intra-day precisions were from 1.5% to 25.4%， and the inter-day precisions were from 0.6% to 23.5%. After correction by the stable-isotope internal standard method， the matrix effects of the 22 target analytes in fetal bovine serum were 83.0%‒119.9%. The developed method was successfully used to detect 22 OUVs in 110 human serum samples. With the exception of 3-benzylidene camphor （3-BC）， 2-（2*H*-benzotriazol-2-yl）-4-methyl-6-（2-propenyl）phenol （UV-9）， 4-methylbenzylidene camphor （4-MBC）， 2，2′-dihydroxy-4-methoxybenzophenone （BP-8）， and 2，4-dihydroxybenzophenone （BP-1）， which were not detected， the remaining 17 substances were detected with overall detection rates of 0.9%–65.5% and the detection levels were <MQL‒11.7 ng/mL. The developed analytical method is simple， convenient， highly sensitive， and is expected to become an effective tool for quantifying 22 OUVs in human serum.

有机紫外吸收剂（organic ultraviolet absorbent， OUVs）是一类能够吸收紫外线的化学物质^［1］^。常见的OUVs可根据化学结构分为多个类别，主要包括对氨基苯甲酸酯类、二苯甲酮类、樟脑衍生物类、肉桂酸酯类、二苯甲酰甲烷类、水杨酸酯类、三嗪类和苯并三唑类等^［2］^。据调查，全球每年约生产1万吨OUVs^［3］^，这些OUVs被广泛用于个人护理产品^［4，5］^和塑料等工业产品中^［6］^。然而，随着这些化学品的广泛使用，OUVs已在水体^［7，8］^、室内灰尘^［3，9］^、室外空气^［10，11］^和土壤^［12］^等多种介质中被检出，甚至还出现在食品包装材料^［13］^和食品中^［14］^。人类暴露于OUVs几乎不可避免，在多个国家的人群研究中^［15］^，已在儿童、孕妇和成年人的血清样本^［16，17］^以及母乳^［18］^和胎盘^［19］^中检测出OUVs。鉴于OUVs的潜在危害及其在环境中的广泛分布，科学界已普遍认识到监测OUVs在人体内暴露水平的必要性。目前，尿液常被作为检测基质用于评估人体内OUVs的暴露水平，而本研究选择血清作为检测基质，主要是因为它能够有效累积脂溶性的OUVs，并准确反映全身循环中的暴露水平。与仅能反映短期暴露的尿液样本相比，血清能够提供更为全面的长期暴露信息。因此，在血清中检测OUVs对于理解健康风险和制定相应干预措施具有重要意义。

为满足对OUVs的监测需求，目前已有少数文献报道了血清中OUVs的分析方法。这些分析方法主要包括气相色谱-串联质谱（GC-MS/MS）技术^［20］^和液相色谱-串联质谱（LC-MS/MS）技术^［21，22］^。GC-MS/MS技术需要对样品进行衍生化处理，过程繁琐且耗时，还可能引入额外的变异因素。此外，也有采用高效液相色谱-光电二极管阵列检测器（HPLC-PDA）的方法^［23］^，但该方法普及程度不高，不适合大面积推广。OUVs的前处理方法主要包括液液萃取法、分散液液萃取法和织物相吸附萃取法。液液萃取法耗时、费力，且消耗大量有机溶剂，对环境和操作人员均不友好，同时通常难以避免目标分析物的损失。织物相吸附萃取法虽然操作步骤相对简单，但在处理大规模样品时，其自动化程度仍有待提高。蛋白质沉淀法主要针对血清中的蛋白质，但对脂质的去除效果有限。脂质的存在可能会干扰色谱的分离性能，并且由于其高黏性和低挥发性，还可能污染色谱柱和质谱检测器，从而增加维护成本。因此，去除脂质不仅有利于提高分析的准确性和灵敏度，还能改善分离效果并保护分析设备。将蛋白质沉淀技术与脂质去除技术相结合，可以获得更为纯净的样品，进而提升分析质量和设备性能。

当前针对血清中OUVs的检测方法，在检测类别和数量上存在一定的局限性。全面了解OUVs的分布和特征对于评估其对人体健康的影响至关重要。因此，本研究旨在对市场上广泛使用的多种OUVs进行系统调研，研究范畴包括5种二苯甲酮（BP）类化合物、5种苯并三唑类化合物、2种肉桂酸酯类化合物及其3种代谢物、2种水杨酸酯类化合物、2种樟脑衍生物类化合物、1种三嗪类化合物、1种二苯甲酰甲烷类化合物以及1种氨基苯甲酸衍生物类化合物。本研究创新性地将蛋白质沉淀技术、高效脂质去除技术与超高效液相色谱-串联质谱（UPLC-MS/MS）结合，建立了一种血清中22种OUVs的检测方法。通过该方法，我们能够更加全面、高效地检测血清中的多种OUVs，并成功用于实际样品的检测。

## 1 实验部分

### 1.1 仪器、试剂与材料

I-Class Plus超高效液相色谱-TQ-XS三重四极杆质谱仪、96孔正压装置（美国Waters公司）；3FCG-11848915E氮吹仪（美国Organomation公司）；Cascada超纯水机（美国波尔公司）；SPSS 27.0统计软件（美国IBM公司）。

22种OUVs标准品：水杨酸辛酯（EHS，纯度99.4%）、甲氧基肉桂酸乙基己酯（EHMC，纯度100%）和2-（2*H*-苯并三唑-2-基）-4-甲基-6-（2-丙烯基）苯酚（UV-9，纯度100%）购于美国AccuStandard公司；2-（2-羟基-5-甲基苯基）苯并三唑（UV-P，纯度99.9%）、2-（5-氯-2*H*-苯三唑-2-基）-6-（1，1-二甲基乙基）-4-甲基苯酚（UV-326，纯度99.9%）、2-（2*H*-苯并三唑-2-基）-4，6-二叔戊基苯酚（UV-328，纯度99.8%）、2-（2′-羟基-5′-叔辛基苯基）苯并三唑（UV-329，纯度98.7%）和2，4-二［4-（2-乙基己氧基）-2-羟基苯基］-6-（4-甲氧基苯基）-1，3，5-三嗪（BEMT，纯度99.3%）购于中国CATO公司；4-甲氧基肉桂酸（4-MCA，纯度99.4%）购于天津阿尔塔公司；二苯甲酮-3（BP-3，纯度98%）购于美国Sigma Aldrich公司；胡莫柳酯（HMS，100.37 μg/mL）、4′-甲氧基苯乙酮（4′-MAP，纯度99.4%）、奥克立林（OC，纯度99.8%）、阿伏苯宗（AVO，纯度99.1%）和3-亚苄基樟脑（3-BC，纯度99.12%）购于德国Dr. Ehrenstorfer公司；二乙氨基羟苯甲酰基苯甲酸己酯（DHHB，纯度98%）、4-甲基苄亚基樟脑（4-MBC，纯度98%）、2-氰基-3，3-二苯基丙烯酸（CDAA，纯度96%）、2，4-二羟二苯甲酮（BP-1，纯度97%）、2，2′，4，4′-四羟基二苯甲酮（BP-2，纯度97%）、4-羟基-二苯甲酮（4-OHBP，纯度97%）和2，2′-羟基-4-甲氧基二苯甲酮（BP-8，纯度97%）均购于加拿大TRC公司。

15种内标：4-MBC-D_4_（纯度98%）、EHS-D_4_（纯度98%）、HMS-D_4_（纯度95%）、EHMC-D_3_（纯度97%）、OC-^13^C_3_（纯度97%）、UV-329-^13^C_6_（纯度95%）、UV-P-D_3_（纯度97%）、AVO-^13^CD_3_（纯度98%）、UV-328-D_12_（纯度97%）、UV-326-D_3_（纯度98%）及BP-1-D_5_、BP-2-D_4_、BP-3-^13^C_6_、BP-8-D_3_和4-OHBP-D_4_（纯度均>97%）均购于加拿大TRC公司。

甲醇和乙腈（LC-MS级）购自德国Merck公司；Anavo-HMR-Lipid磷脂去除板（10 mg）购于北京纳鸥科技有限公司；Biotage ISOLUTE^®^ PLD+蛋白沉淀除磷脂板（40 mg）和ISOLUTE^®^ SLE+固相支撑液液萃取板（400 μL）购于瑞典Biotage AB公司；EMR-Lipid磷脂去除板（50 mg）购于美国Agilient公司；Phree磷脂去除板（30 mg）购于美国Phenomenex公司；96孔收集板（800 μL/2 mL）购于美国Waters公司。

### 1.2 溶液配制

22种OUVs混合标准储备液和混合内标储备液的配制参考文献［^24^］。22种OUVs混合标准储备液的质量浓度为1 000 ng/mL，混合内标储备液的质量浓度为1 000 ng/mL，其中BP-3-D_3_的质量浓度为4 000 ng/mL。实验开始前，将混合内标储备液稀释20倍，制备成质量浓度为50 ng/mL的混合内标工作液（其中BP-3-D_3_的质量浓度为200 ng/mL）。接着，用甲醇将22种OUVs混合标准储备液稀释成系列质量浓度（0、1、2.5、5、10、25、50、100、500 ng/mL）的混合标准溶液。最后，分别将50 μL各质量浓度下的混合标准溶液与150 μL混合内标工作液混合，用20%甲醇水溶液稀释至500 μL，制备成系列质量浓度（0、0.1、0.25、0.5、1、2.5、5、10、25、50 ng/mL）的混合标准工作溶液。所有标准溶液均保存于4 °C冰箱中，以确保其稳定性和有效性。

### 1.3 样品采集

人血清样本采集自海南、黑龙江和西藏地区的健康志愿者。采集完毕后，将血清样品分装至1.5 mL样品管中，并使用干冰运输至实验室，随后在‒80 ℃条件下保存，直至进行分析。本研究已获得中国疾病预防控制中心环境与健康相关产品安全所伦理委员会的批准（批准文件号：No. 201904）。

### 1.4 样品前处理

取50 μL解冻至室温的血清样本加入到HMR-Lipid磷脂去除板中，加入50 μL 50 ng/mL的混合内标工作液（其中BP-3-D_3_的质量浓度为200 ng/mL），再加入200 μL蛋白质沉淀溶剂乙腈，静置5~10 min。随后，通过正压过滤的方式收集洗脱液，在过滤过程中将压力（3 Pa）逐渐增大至7 Pa，以确保液体连续且缓慢地滴落。重复上述操作，继续向HMR-Lipid磷脂去除板中添加200 μL乙腈，并进行正压过滤，随后将两次收集的洗脱液合并，在25 °C条件下进行氮吹，直至溶剂完全挥发。最后，使用50 μL甲醇复溶，进行UPLC-MS/MS分析。

### 1.5 分析条件

#### 1.5.1 色谱条件

色谱柱：Acquity BEH C_18_柱（100 mm×2.1 mm，1.7 μm）；流动相1：A相为纯水，B相为甲醇，用于检测BP类（BP-1、BP-2、BP-3、BP-8和4-OHBP）OUVs和AVO；流动相2：A相为0.1%氨水，B相为甲醇，用于检测其余16种OUVs；柱温箱温度：40 °C；进样量：5 μL。梯度洗脱程序：0~5.0 min，30% B~60% B；5.0~5.1 min，60% B~80% B；5.1~22.0 min，80% B~100% B；22.0~22.1 min，100% B~70% B；22.1~25.0 min，70% B~30% B。

#### 1.5.2 质谱条件

离子源：电喷雾电离（ESI）源，正、负离子扫描，多反应监测（MRM）模式；碰撞气：氩气；毛细管电压：2.46 kV；离子源温度：150 °C；去溶剂温度：500 °C。22种OUVs及15种内标的详细质谱参数见表1。

**表 1:** 22种OUVs及15种内标的质谱参数

Compound	Retention time/min	Parent ion （*m/z*）	Daughter ions （*m/z*）	CV/V	CEs/eV	Internal standard
4′-Methoxyacetophenone （4′-MAP）	5.57	151.1	42.9^*^， 93.9	18	12， 20	EHMC-D_3_
2，2′，4，4′-Tetrahydroxybenzophenone （BP-2）	5.59	245.0	91.0， 135.0^*^	‒10	‒28， ‒16	BP-2-D_4_
4-Hydroxybenzophenone （4-OHBP）	6.67	196.9	91.9^*^， 120.1	‒10	‒26， ‒24	4-OHBP-D_4_
2，2′-Dihydroxy-4-methoxybenzophenone （BP-8）	6.72	243.9	93.1^*^， 123.5	‒20	‒22， ‒16	BP-8-D_3_
2，4-Dihydroxybenzophenone （BP-1）	6.89	212.9	91.0， 135.0^*^	‒16	‒24， ‒20	BP-1-D_5_
Oxybenzone （BP-3）	7.42	227.1	167.0， 211.2^*^	‒8	‒34， ‒16	BP-3-D_3_
3-Benzylidene camphor （3-BC）	8.29	241.2	91.0^*^， 157.1	10	32， 16	4-MBC-D4
2-（2-Hydroxy-5-methylphenyl）benzotriazole （UV-P）	8.65	226.1	107.0， 120.0^*^	12	20， 18	UV-P-D_3_
4-Methylbenzylidene camphor （4-MBC）	9.07	259.1	97.1， 105.0^*^	28	18， 26	4-MBC-D_4_
Diethylaminohydroxybenzoylhexyl benzoate （DHHB）	9.57	398.3	148.9^*^， 233.2	30	18， 10	OC-^13^C_3_
Octocrylene （OC）	10.77	362.5	232.3^*^， 250.1	20	20， 20	OC-^13^C_3_
2-Cyano-3，3-diphenylpropenoic acid （CDAA）	10.78	250.0	201.0， 207.0^*^	20	25， 15	OC-^13^C_3_
Avobenzone （AVO）	11.75	310.9	134.9， 161.2^*^	20	20， 20	AVO-^13^CD
Ethylhexyl methoxycinnamate （EHMC）	11.76	290.9	160.8^*^， 179.1	16	12， 12	EHMC-D_3_
4-Methoxycinnamic acid （4-MCA）	11.77	178.9	132.9^*^， 117.9	18	18， 24	EHMC-D_3_
Ethylhexyl salicylate （EHS）	11.96	249.0	93.0， 249.0^*^	‒15	‒5， ‒22	EHS-D_4_
Homosalate （HMS）	12.04	261.0	93.0， 137.0^*^	‒18	‒18， ‒18	HMS-D_4_
2-（2*H*-Benzotriazol-2-yl）-4-methyl-6-（2-propenyl）phenol （UV-9）	12.06	266.3	119.0^*^， 147.0	15	18， 16	EHS-D_4_
2-（2′-Hydroxy-5′-（1-1-3-3-tetramethylbutyl）phenyl）benzotriazole （UV-329）	12.46	324.2	57.0， 212.1^*^	4	26， 22	UV-329-^13^C_6_
2-（5-Chlor-2*H*-benzotriazol-2-yl）-6-（1，1-dimethylethyl）-4-methyl-phenol （UV-326）	13.04	316.1	57.0， 106.9^*^	50	24， 24	UV-326-D_3_
2-（2*H*-Benzotriazol-2-yl）-4，6-ditertpentylphenol （UV-328）	13.26	352.2	71.1， 282.3^*^	22	25， 21	UV-328-D_12_
2，4-Bis［4-（2-ethylhexyloxy）-2-hydroxyphenyl］-6-（4-methoxyphenyl）-1，3，5-triazine （BEMT）	19.29	628.3	136.0^*^， 404.1	14	54， 42	EHMC-D_3_
BP-2-D_4_	5.56	249.1	110.9	‒16	‒4	/
4-OHBP-D_4_	6.66	200.9	95.8	‒30	‒6	/
BP-8-D_3_	6.71	246.1	125.9	‒20	‒18	/
BP-1-D_5_	6.89	218.1	134.9	‒20	‒36	/
BP-3-D_3_	7.42	230.0	166.9	‒16	‒44	/
UV-P-D_3_	8.63	229.0	81.9	2	26	/
4-MBC-D_4_	9.02	259.1	98.1	26	18	/
OC-^13^C_3_	10.71	364.9	252.9	40	18	/
EHMC-D_3_	11.76	293.9	181.9	14	8	/
AVO-^13^CD	11.79	315.1	161.0	6	24	/
EHS-D_4_	11.95	252.9	140.9	‒20	‒18	/
HMS-D_4_	12.03	264.9	140.9	‒16	‒18	/
UV-329-^13^C_6_	12.44	330.2	56.9	56	24	/
UV-326-D_3_	13.01	319.0	263.0	4	18	/
UV-328-D_12_	13.22	364.3	289.3	2	24	/

CV： cone voltage； CEs： collision energies； * quantitative ion； /： no value.

### 1.6 质量控制

为确保实验结果的准确性和可重复性，我们依据参考文献［^24^］对实验过程中的空白进行了仔细筛查。筛查结果发现，聚乙烯（PE）材质的耗材中存在UV-P和UV-326等化合物空白值不稳定的情况。因此，我们优先选择使用聚丙烯（PP）材质的耗材，并对每批次耗材进行空白值评估，确保在无空白值干扰情况下使用。此外，每次实验开始前和结束后，我们都对实验环境进行彻底清洁，避免在后续实验中引入目标分析物。

## 2 结果与讨论

### 2.1 液相色谱条件优化

#### 2.1.1 流动相的优化

本研究以Acquity BEH C_18_色谱柱作为分析柱，比较了甲醇-水（中性）、甲醇-0.1%氨水（碱性）和甲醇-0.1%甲酸水（酸性）3种流动相体系对目标化合物色谱峰形和响应值的影响。实验结果表明，大部分OUVs在甲醇-0.1%氨水流动相体系中的质谱响应值达到最高；其中，EHS和HMS不但在甲醇-0.1%氨水流动相中的响应值较高，且色谱峰形也较好。在甲醇-水流动相体系中，AVO和BP类OUVs的色谱峰形表现最佳，且他们的响应值也明显高于另外两种流动相体系下的结果。因此，为了全面检测22种目标化合物，本研究采用甲醇-水流动相（中性）检测BP类OUVs和AVO，用甲醇-0.1%氨水（碱性）流动相检测其余16种OUVs。

#### 2.1.2 色谱柱的选择

在优化后的流动相体系下，比较Acquity UPLC HSS T_3_（100 mm×2.1 mm，1.8 μm）、Acquity BEH C_18_（100 mm×2.1 mm，1.7 μm）和Acquity BEH Phenyl（100 mm×2.1 mm，1.7 μm）3种色谱柱对22种OUVs的分离效果。结果显示，与其他两种色谱柱相比，C_18_柱对22种OUVs的分离效果更佳。尽管EHS在T_3_柱上的响应值略高于C_18_柱，但22种OUVs在T_3_柱上的出峰时间更长，且CDAA在T_3_柱上几乎没有出峰。同样地，采用Phenyl柱进行分离时，CDAA、EHMC和4-MCA的出峰效果较差。因此，本实验选择Acquity BEH C_18_柱对这22种目标分析物进行分离。在最优流动相和色谱柱条件下，22种OUVs的MRM色谱图如图1所示。

**图1:**
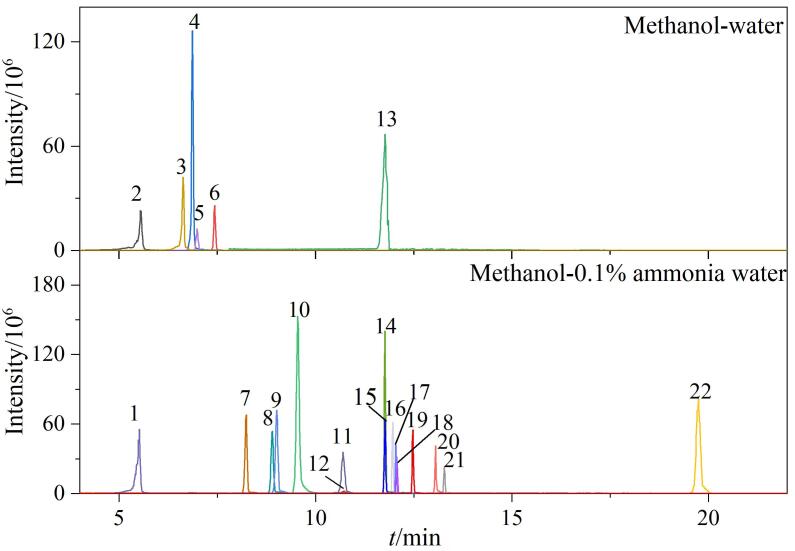
22种OUVs混合标准溶液（50 ng/mL）的MRM色谱图

### 2.2 前处理条件优化

#### 2.2.1 前处理方式的选择

本研究分别比较了HMR-Lipid磷脂去除板（10 mg）、ISOLUTE^®^ PLD+蛋白沉淀除磷脂板（40 mg）、ISOLUTE^®^ SLE+固相支撑液液萃取板（400 μL）、EMR-Lipid磷脂去除板（50 mg）和Phree磷脂去除板（30 mg）等5种前处理方式对血清样品中磷脂的去除效果。在加标水平为10 ng/mL时，以目标化合物的绝对回收率（按照文献［^25^］方法计算）为指标，考察5种前处理方式对22种OUVs的净化效果，结果如图2所示。结果表明，在使用SLE+固相支撑液液萃取板和Phree磷脂去除板时，大部分目标化合物的绝对回收率均低于50%。采用PLD+蛋白沉淀除磷脂板进行前处理时，BEMT、BP-8、EHS和4′-MAP的绝对回收率也同样低于50%。在使用HMR-Lipid磷脂去除板进行前处理时，各目标化合物的绝对回收率均高于80%，并且普遍高于EMR-Lipid磷脂去除板。这可能是因为HMR-Lipid磷脂去除板所采用的无机锆材料对磷脂具有较高的特异性吸附能力，使得其除脂效率高于其他填料。因此，本研究最终采用HMR-Lipid磷脂去除板（10 mg）进行血清样本的前处理。

**图2:**
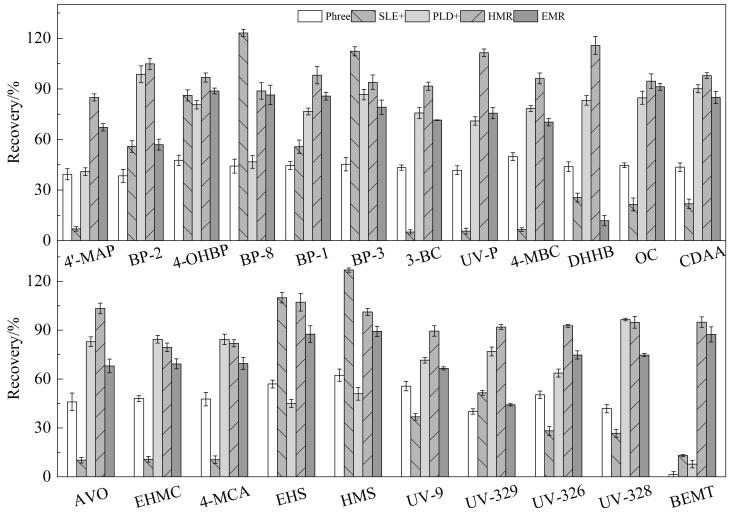
不同前处理方式对22种OUVs绝对回收率的影响（*n*=5）

#### 2.2.2 上样体积的优化

在确定了前处理方式后，我们对血清样本的上样体积进行了优化。在沉淀溶剂种类、体积及沉淀次数一致的情况下，实验分别考察了50 μL和100 μL的上样体积对22种OUVs响应值的影响。实验结果显示，当上样体积为50 μL时，各目标化合物的响应值均高于100 μL上样体积时的响应值。进一步增加样品上样量并未提升各目标化合物的响应值。推测其原因，可能是100 μL的上样体积超出了填料的承载负荷，进而影响了检测效果。因此，本研究选择50 μL作为优化后的上样体积。

#### 2.2.3 沉淀溶剂的选择

通常使用甲醇或乙腈作为血清样品的蛋白质沉淀溶剂。在上样体积、沉淀溶剂体积及沉淀次数一致的情况下，分别使用甲醇和乙腈对血清样品进行蛋白质沉淀，比较两种沉淀溶剂对22种OUVs绝对回收率的影响。当使用甲醇作为沉淀溶剂时，22种目标化合物的绝对回收率为39.4%~77.6%；改用乙腈后，22种目标化合物的绝对回收率均明显提高（82.0%~115.8%）。这种差异可能归因于甲醇沉淀法采用柱外沉淀操作，在将上清液转移至小柱的过程中，目标物可能会发生不同程度的损失。因此，本研究选择乙腈作为蛋白质沉淀溶剂。

#### 2.2.4 沉淀溶剂体积的优化

在上样体积、沉淀溶剂种类及沉淀次数一致的情况下，实验考察了不同体积（150 μL和200 μL）的沉淀溶剂对22种OUVs响应值的影响。结果表明，与150 μL乙腈相比，使用200 μL乙腈时，22种OUVs的响应值更高，因此确定乙腈的体积为200 μL。

#### 2.2.5 沉淀次数的选择

在上样体积、沉淀溶剂种类及体积一致的情况下，实验对沉淀次数进行了优化，分别考察不同沉淀次数（1、2、3和4次）对22种OUVs响应值的影响。结果表明，与其他沉淀次数相比，当沉淀次数为2次时，13种目标化合物（DHHB、BEMT、4-MCA、AVO、EHMC、BP-3、BP-8、UV-326、UV-9、4-MAP、UV-329、CDAA和BP-1）的响应值达到最高；但若继续增加沉淀次数，会造成EHMC、BP-2、BP-3和AVO等化合物的损失。因此，本实验最终选择沉淀2次（每次使用200 μL乙腈）。

### 2.3 基质效应

以空白胎牛血清为基质，按1.4节方法进行前处理，用空白基质提取液配制系列质量浓度（0.1、0.25、0.5、1、2.5、5、10、25、50 ng/mL）的基质匹配混合标准溶液，同时配制相同质量浓度的溶剂混合标准溶液。按照文献［^26^］方法，利用基质匹配标准曲线与溶剂标准曲线斜率的比值来评估基质效应（ME）。当ME为80%~120%时，判断为弱基质效应；当ME为50%~80%或120%~150%时，判断为中等基质效应；当ME<50%或>150%时，判断为强基质效应。如图3a所示，4′-MAP、UV326和4-OHBP表现为强基质效应（175.4%~559.9%），BP-2、BP-3、AVO、DHHB、UV-328、4-MCA、UV-P、3-BC、4-MBC、OC、BEMT、BP-8和UV-9表现为中等基质效应（64.1%~139.5%），CDAA、EHMC、UV-329、BP-1、EHS和HMS表现为弱基质效应（93.9%~118.8%）。当采用稳定同位素内标法校正之后，22种OUVs的基质效应均表现为弱基质效应（83.0%~119.9%）（图3b）。

**图3:**
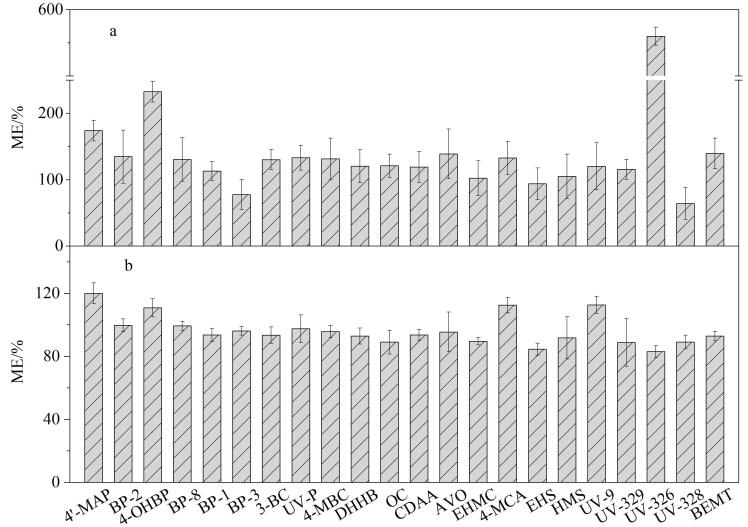
采用稳定同位素内标法校正（a）前、（b）后22种OUVs的基质效应（*n*=3）

### 2.4 方法学评价

#### 2.4.1 线性范围、方法检出限和方法定量限

配制系列质量浓度（0.1、0.25、0.5、1、2.5、5、10、25、50 ng/mL）的混合标准工作溶液，按1.5节条件进行测定。以目标化合物与相应内标的定量离子峰面积之比为纵坐标（*Y*），目标化合物的质量浓度为横坐标（*X*， ng/mL），绘制标准曲线。对于有过程空白的目标化合物（4-MCA、UV-P、EHMC、AVO、UV-326、OC、DBT、BP-1、BP-8、BP-2和HMS），分别进行5次过程空白实验，并用标准曲线对目标化合物进行定量分析，计算5次过程空白响应值浓度的平均值（ $\bar{x}$ ）及标准偏差（SD）。根据 $\bar{x}$ +3SD确定方法检出限（MDL），根据 $\bar{x}$ +10SD确定方法定量限（MQL）。对于不含过程空白的目标化合物（3-BC、CDAA、4-MBC、UV-329、UV-328、DHHB、BP-3、4′-MAP、4-OHBP、EHS和UV-9），采用空白血清低水平加标（0.2 ng/mL）实验，经前处理后上机测定。计算各目标化合物5次测定结果的3SD，作为MDL；计算各目标化合物5次测定结果的10SD，作为MQL。如表2所示，22种目标化合物在各自的线性范围内线性关系良好，相关系数（*r*）均≥0.999 3，MDL为0.02~0.48 ng/mL，MQL为0.02~1.60 ng/mL。

**表 2:** 22种OUVs的线性方程、线性范围、相关系数、方法检出限和方法定量限

Compound	Linear equation	Linear range/（ng/mL）	*r*	MDL/（ng/mL）	MQL/（ng/mL）
EHS	*Y*=0.9996*X*+0.0653	0.33‒50	0.9996	0.10	0.33
HMS	*Y*=0.0413*X*+0.0291	0.17‒50	0.9997	0.08	0.17
EHMC	*Y*=0.0279*X*+0.0012	0.17‒50	0.9999	0.09	0.17
4′-MAP	*Y*=0.1410*X*+0.0509	0.49‒50	0.9998	0.15	0.49
4-MCA	*Y*=0.0492*X*+0.0059	0.12‒50	0.9997	0.10	0.12
AVO	*Y*=0.1362*X*+0.0243	0.05‒50	0.9999	0.02	0.05
OC	*Y*=0.0351*X*+0.0003	0.37‒50	0.9997	0.20	0.37
CDAA	*Y*=0.0001*X*+0.0003	1.60‒50	0.9997	0.48	1.60
4-MBC	*Y*=0.3431*X*+0.1090	0.26‒50	0.9999	0.08	0.26
3-BC	*Y*=0.4006*X*+0.0419	0.09‒50	0.9996	0.03	0.09
UV-P	*Y*=0.1367*X*+0.0364	0.27‒50	0.9999	0.12	0.27
UV-9	*Y*=1.5741*X*+0.2905	0.01‒50	0.9993	0.06	0.19
UV-326	*Y*=0.0154*X*+0.0095	0.25‒50	0.9998	0.10	0.25
UV-328	*Y*=0.4156*X*+0.2289	0.15‒50	0.9996	0.04	0.15
UV-329	*Y*=0.2146*X*+0.1733	0.23‒50	0.9995	0.07	0.23
BP-1	*Y*=0.0482*X*+0.0128	0.12‒50	0.9999	0.08	0.12
BP-2	*Y*=0.0992*X*+0.0121	0.02‒50	0.9999	0.02	0.02
BP-3	*Y*=1.2280*X*+0.2927	0.09‒50	0.9999	0.03	0.09
BP-8	*Y*=0.0041*X*+0.0121	0.15‒50	0.9999	0.13	0.15
4-OHBP	*Y*=0.0535*X*+0.1349	0.30‒50	0.9999	0.09	0.30
BEMT	*Y*=0.0492*X*+0.0384	0.32‒50	0.9996	0.14	0.32
DHHB	*Y*=0.8267*X*+0.5613	0.11‒50	0.9996	0.03	0.11

*Y*： peak area ratio of target compound to the corresponding internal standard； *X*： mass concentration， ng/mL.

#### 2.4.2 回收率与精密度

在空白胎牛血清中分别添加低、中、高3个水平（1、5、25 ng/mL）的混合标准工作溶液，进行加标回收试验。每个加标水平样品在1 d内平行测定5次，计算加标回收率和日内精密度；在25 ng/mL加标水平下连续测定5 d，计算日间精密度。结果显示，在3个加标水平下，22种目标化合物的加标回收率和日内精密度分别为79.9%~136.1%和1.5%~25.4%；在25 ng/mL加标水平下，日间精密度为0.6%~23.5%（见附表1，https://www.chrom-China.com）。结果表明，该方法具有良好的回收率和精密度，能够用于血清样本中22种OUVs的定量分析。

### 2.5 实际样品测定

为验证方法的实用性，将该方法应用于人体生物监测项目中110份血清样品中OUVs的测定，并采用SPSS 27.0统计软件进行分析。如表3所示，在110份血清样品中，除3-BC、UV-9、4-MBC、BP-8和BP-1未检出外，其余17种OUVs均有检出，检出率为0.9%~65.5%，检出水平为<MQL~11.7 ng/mL。在17种检出的OUVs中，检出率位于前3的OUVs分别是UV-P、EHS和4-MCA，其中UV-P的检出水平最大值为9.4 ng/mL。上述结果表明，人群普遍暴露于OUVs，需引起关注。

**表 3:** 22种OUVs在人血清中的测定结果

Compound	*N*	DR/%	Minimum/（ng/mL）	Maximum/（ng/mL）	Percentile levels /（ng/mL）		
					5th	50th	95th
4′-MAP	17	15.5	<MQL	<MQL	<MQL	<MQL	<MQL
4-MCA	31	28.2	<MQL	1.2	<MQL	<MQL	0.4
UV-P	72	65.5	<MQL	9.4	<MQL	0.2	3.0
CDAA	9	8.2	<MQL	11.7	<MQL	<MQL	2.7
EHMC	6	5.5	<MQL	0.9	<MQL	<MQL	0.2
UV-326	27	24.6	<MQL	8.7	<MQL	<MQL	<MQL
UV-329	7	6.4	<MQL	1.4	<MQL	<MQL	0.5
UV-328	1	0.9	<MQL	<MQL	<MQL	<MQL	<MQL
OC	12	10.9	<MQL	9.8	<MQL	<MQL	2.6
DHHB	16	14.6	<MQL	0.1	<MQL	<MQL	<MQL
BEMT	3	2.7	<MQL	0.6	<MQL	<MQL	<MQL
EHS	49	44.6	<MQL	5.8	<MQL	<MQL	4.3
HMS	10	9.1	<MQL	0.6	<MQL	<MQL	<MQL
AVO	29	26.4	<MQL	2.7	<MQL	<MQL	<MQL
4-OHBP	1	0.9	<MQL	<MQL	<MQL	<MQL	<MQL
BP-3	12	10.9	<MQL	0.3	<MQL	<MQL	<MQL
BP-8	0	0	/	/	/	/	/
BP-2	1	0.9	<MQL	<MQL	<MQL	<MQL	<MQL
4-MBC	0	0	/	/	/	/	/
UV-9	0	0	/	/	/	/	/
BP-1	0	0	/	/	/	/	/
3-BC	0	0	/	/	/	/	/

*N*： number of positive samples； DR： detection rate.

### 2.6 方法比较

目前能够同时检测人血清中多种OUVs的方法较为有限，经文献调研，最多可同时测定7种OUVs。将本文所建方法与文献报道方法进行比较，结果如表4所示。与文献方法相比，本方法具有以下优势：（1）能够同时分析22种OUVs，数量上超过现有方法；（2）所需样品体积少，仅需50 μL血清，有助于节约生物样本资源并提高大批量样品测定的可行性；（3）有机溶剂使用量少（仅需400 μL），降低了实验人员的风险，增强了操作的安全性；（4）本方法的MDL较低，能够满足小体积血清样本中微量OUVs的检测需求。

**表 4:** 本方法与文献方法的比较

Pretreatment	Numbers of compounds	Sample volume/mL	Solvent volume/mL	Analytical method	MDL/（ng/mL）	Ref.
LLE	7	1	5	LC-MS/MS	-	［^16^］
PPT	6	0.1	0.1	LC-MS/MS	0.08‒0.27	［^21^］
DLLME	4	1	1	UHPLC	-	［^27^］
-	3	0.15	0.45	LC-MS/MS	1.1‒6.5	［^28^］
DLLME	1	1	2.7	GC-AEI-MS/MS	0.1	［^20^］
PPT	5	0.15	0.5	HPLC-MS/MS	0.1‒0.4	［^22^］
PPT+ELR	22	0.05	0.4	UPLC-MS/MS	0.02‒0.48	this study

PPT： protein precipitation technology； ELR： efficiency lipid removal technology； -： not mentioned； AEI： advanced electron ionization.

## 3 结论

本研究将蛋白质沉淀技术、高效脂质去除技术与UPLC-MS/MS技术结合，建立了一种同时测定血清中22种OUVs的方法。该方法展现出优异的灵敏度、准确度和精密度，这些优异的性能指标确保了该方法在实际应用中的可靠性和有效性，使之成为研究和监测人体内OUVs暴露水平的重要工具。
